# Three-Tier Prognostic Index in Young Adults With Advanced Gastric Cancer

**DOI:** 10.3389/fonc.2021.667655

**Published:** 2021-09-10

**Authors:** Guang-Liang Chen, Yan Huang, Wen Zhang, Xu Pan, Wan-Jing Feng, Xiao-Ying Zhao, Xiao-Dong Zhu, Wen-Hua Li, Mingzhu Huang, Zhi-Yu Chen, Wei-Jian Guo

**Affiliations:** ^1^Department of Medical Oncology, Fudan University Shanghai Cancer Center, Shanghai, China; ^2^Department of Oncology, Shanghai Medical College Fudan University, Shanghai, China; ^3^Department of Oncology and Chemotherapy, Red Cross Hospital of Yulin City, Yulin, China; ^4^Department of Medical Oncology, Yixing Traditional Chinese Medicine Hospital, Wuxi, China

**Keywords:** advanced gastric cancer, young adults, prognostic factors, albumin, neutrophil–lymphocyte ratio

## Abstract

**Purpose:**

To characterize clinical features and identify baseline prognostic factors for survival in young adults with advanced gastric cancer (YAAGC).

**Materials and Methods:**

A total of 220 young inpatients (age less than or equal to 40 years) with an initial diagnosis of advanced gastric cancer were retrospectively enrolled in this study.

**Results:**

Of a consecutive cohort of 220 patients with YAAGC, the median overall survival (OS) time was 16.3 months. One-year survival rate was 43.6% (95% CI: 36.5 to 50.7). In this cohort, a female (71.4%, *n* = 157) predominance and a number of patients with poorly differentiated tumors (95.9%, *n* = 211) were observed. In the univariate analysis, OS was significantly associated with neutrophil–lymphocyte ratio (NLR) (≥3.12), hypoproteinemia (<40 g/L), presence of peritoneal or bone metastases, and previous gastrectomy of primary tumor or radical gastrectomy. In multivariate Cox regression analysis, hypoproteinemia [hazard ratio (HR) 1.522, 95% CI 1.085 to 2.137, *p* = 0.015] and high NLR level (HR 1.446, 95% CI 1.022 to 2.047, *p* = 0.021) were two independent poor prognostic factors, while previous radical gastrectomy was associated with a favorable OS (HR 0.345, 95% CI 0.205 to 0.583, *p* = 0.000). A three-tier prognostic index was constructed dividing patients into good-, intermediate-, or poor-risk groups. Median OS for good-, intermediate-, and poor-risk groups was 36.43, 17.87, and 11.27 months, respectively.

**Conclusions:**

Three prognostic factors were identified, and a three-tier prognostic index was devised. The reported prognostic index may aid clinical decision-making, patient risk stratification, and planning of future clinical studies on YAAGC.

## Introduction

Gastric cancer (GC) is an aggressive malignancy with significant prevalence and mortality rate in Asia (1, 2). Young adults with GC are regarded as a different clinical entity from carcinogenesis to prognosis (1). The OS of GC in young adults remains poor (1–5). Considering a significant loss of life-years in young patients with GC, decreasing GC mortality needs more extensive studies on this disease.

Clinical stage and treatment are two strong predictors of OS in young patients with GC (2, 5–8). Despite many attempts to characterize the clinical differences between younger and older people with GC (9–12), few studies focused on young adults with GC who were initially diagnosed with advanced GC (YAAGC). One believes that young patients with less comorbidity can tolerate more aggressive treatment (1, 2, 7); however, the prognostic factors are poorly understood. The survival benefit of early detection of GC in young people has come to a consensus (1, 3–6, 8); however, near-universal findings in young patients with GC have seen a female predominance, higher frequency of advanced lesions, and poor-differentiated tumors at presentation in comparison with older patients (1, 3–6, 8). Surgical resection (radical or palliative gastrectomy) is often performed for patients with potentially resectable lesions in practice, which is associated with a favorable outcome in advanced GC (13). Nevertheless, the role of survival benefits after surgical resection remains unknown in general treatment practice for advanced GC in young adults. In addition, laboratory findings (14) such as alkaline phosphatase (ALP) and hemoglobin (Hb), and some well-known prognostic markers (15–17), such as neutrophil–lymphocyte ratio (NLR), still need to be validated in the population of YAAGC.

In this study, we aimed to identify baseline patient- or tumor-related prognostic factors and to devise a prediction model for survival and risk stratification in a large sample size of YAAGC. The devised applicable prognostic index for YAAGC would be valuable for assessing survival prognosis of individual patients, aiding in risk stratification, and guiding decisions for optimal treatment strategies.

## Patients and Methods

### Participants and Study Design

**Figure 1:**
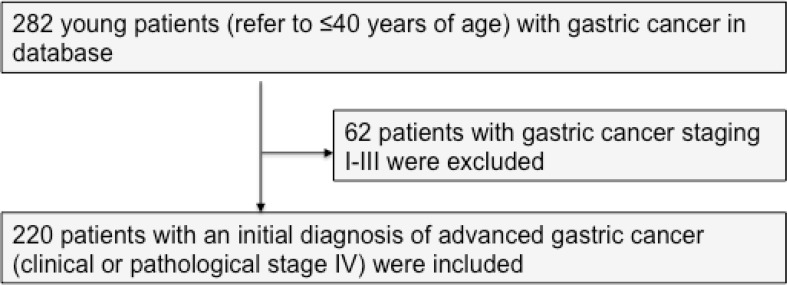
CONSORT diagram.

Between January 2006 and December 2019, a total of 282 young patients (age less than or equal to 40 years) with GC were treated in the Department of Medical Oncology, Fudan University Shanghai Cancer Center (FUSCC). Previously untreated, unresectable, locally advanced, or metastatic adenocarcinoma of the stomach and gastro-esophageal junction was defined as advanced GC. According to the eighth edition of the AJCC/TNM classification issued in 2018, a cohort of 220 patients with an initial diagnosis of advanced GC and complete data were included in this study (**Figure 1**). Two hundred and six patients and 14 patients had stage IVB and IVA disease, respectively. One hundred forty-five young patients were diagnosed and treated in our hospital initially. One patient with liver oligometastasis underwent surgery after chemotherapy. Data were collected retrospectively. An independent researcher who was not involved in the care of patients conducted the construction of the database. Electronic medical records were used to obtain demographic variables (age and gender), clinical variables, laboratory values, and medications. Mortality data and timing of death were obtained from the Department of Cancer Prevention, FUSCC. Eighteen patients (8.2%) were considered lost to follow-up if the last visit was >6 months before the end of the study. The primary outcome was OS that was measured as the time from the diagnosis of advanced GC disease to death, date of last follow-up, or December 30, 2019. This study was conducted in accordance with the ethical principles originating in the Declaration of Helsinki, good clinical practices, and all applicable laws and regulations. The Institutional Review Board of FUSCC approved the study.

### Statistical Analysis

The description of continuous variables and categorical variables is indicated in tables. Continuous variables with normal distribution were compared with the analysis of *t*-tests, while those with non-normal distribution were assessed with nonparametric tests; categorical variables were compared with the chi-square test. Univariate and multivariate Cox regression models were used to assess the association between clinical or laboratory variables and the primary outcome. We reported adjusted hazard ratios (HRs) with their 95% confidence intervals (CIs). The Kaplan–Meier method was used to estimate the overall survival from the time of diagnosis in each group. Differences between the survival curves in both groups were analyzed by the log-rank test. The survival curves were plotted in the software of GraphPad Prism 8.

The construction of the prognostic model started with a univariate assessment of the prognostic effect of each factor. Multivariate analysis was then performed using stepwise Cox proportional hazards regression modeling (entry and exit significance level = 0.01). Then, the final prognostic factors were identified based on a multivariable Cox model. Based on the relative magnitude of each factor’s effect on OS (i.e., HR), a prognostic index was devised and grouped into three levels: good, intermediate, and high risk. A two-sided *p*-value of less than 0.05 was considered significant, and 95% CIs were quoted. All statistical analyses were two-sided and conducted using SPSS version 24.0 for Windows.

## Results

**Figure 2:**
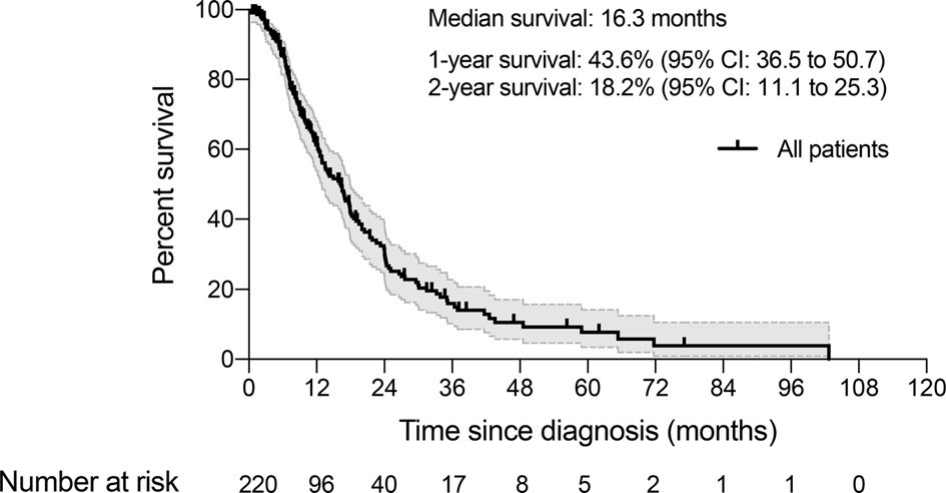
Overall survival for the whole group (*N* = 220).

Between January 2006 and December 2019, we identified a consecutive cohort of 282 young inpatients with GC treated at our institution. After the exclusion criteria were applied, a total of 220 YAAGC patients were included in the analysis (**Figure 1**). After a median of 10.5 months follow-up, 143 (65%) patients died (**Figure 2**). The estimated median OS time was 16.3 months, ranging from 0.5 to 102.7 months. One-year survival and 2-year survival rate was 43.6% (95% CI: 36.5 to 50.7) and 18.2% (95% CI: 11.1 to 25.3), respectively. **Figure 2** shows the OS for the whole group.

**Table 1** summarizes patient baseline characteristics and the results of the univariate analyses for patient- and tumor-related factors. There was a female predominance (71.4%, *n* = 157) in young patients with advanced GC. One-fifth of the patients (*n* = 46) reported a family history of any cancer (*p* = 0.070). Few patients presented with a poor performance status (ECOG ≥2) at admission. The median NLR ratio was 3.12 (range 0.81 to 21.33). A significant number of this population included patients with peritoneal metastasis (60.5%, *n* = 133), poorly differentiated tumors (95.9%, *n* = 211), and bone metastasis (12.7%, *n* = 28). Indeed, high NLR level (≥3.12), hypoproteinemia (albumin < 40 g/L), presence of peritoneal or bone metastases, and previous gastrectomy of primary site or radical gastrectomy were significant for OS in univariate analyses.

**Table 1:** Patient characteristics and univariate analysis.

Characteristics	Overall (*N* = 220)		Univariate Cox model		
	No. of patients	%	HR	95% CI	*p*-value
Female	157	71.4	1.222	0.835 to 1.788	0.301
Married	201	91.4	0.715	0.393 to 1.300	0.272
Family history	46	20.9	1.449	0.970 to 2.165	0.070
Smoker	27	12.3	0.798	0.459 to 1.388	0.425
EOCG performance status ≥ 2	13	5.9	0.833	0.337 to 2.059	0.692
Blood Infusion history	27	12.3	1.058	0.607 to 1.845	0.842
Neutrophil–lymphocyte ratio ≥ 3.12	110	50	1.855	1.324 to 2.600	0.000
Platelets ≥ 350 × 10^9^/L	39	17.7	0.971	0.597 to 1.579	0.906
Hemoglobin < 10 g/L	54	25.9	1.007	0.687 to 1.476	0.972
Alkaline phosphatase > 135 U/L	25	11.4	1.446	0.855 to 2.445	0.168
Albumin < 40 g/L	105	47.7	1.630	1.169 to 2.272	0.004
Lactate dehydrogenase ≥ 260 IU/L	41	18.6	1.067	0.671 to 1.696	0.784
Poor tumor differentiation	211	95.9	1.566	0.640 to 3.833	0.326
Peritoneal metastases	133	60.5	1.778	1.256 to 2.517	0.001
Liver metastases	40	18.2	0.674	0.408 to 1.115	0.124
Bone metastases	28	12.7	1.722	1.045 to 2.838	0.033
Previous gastrectomy of primary site	50	22.7	0.407	0.269 to 0.615	0.000
Radical gastrectomy	33	15.0	0.314	0.188 to 0.524	0.000
Palliative chemotherapy	211	95.9	0.975	0.397 to 2.392	0.956

HR, hazard ratio; ECOG, Eastern Cooperative Oncology Group.

The final multivariable stepwise Cox regression with age, gender, and all significant univariate predictors identified three independent prognostic factors (**Table 2**). Although abnormally low blood levels of albumin (HR 1.522, 95% CI 1.085 to 2.137, *p* = 0.015) and abnormally high levels of NLR (HR 1.446, 95% CI 1.022 to 2.047, *p* = 0.021) were two independent predictive factors of poor prognosis, a previous radical gastrectomy was associated with a significant OS benefit (HR 0.345, 95% CI 0.205 to 0.583, *p* = 0.000).

**Table 2:** Multivariable Cox regression analysis.

Factors	Hazard Ratio	95% CI	*p*-value
Albumin < 40 g/L	1.522	1.085 to 2.137	0.015
NLR ≥ 3.12	1.446	1.022 to 2.047	0.021
Radical gastrectomy			
Yes	1	–	–
No	2.895	1.716 to 4.884	0.000

CI, confidence intervals; NLR, neutrophil–lymphocyte ratio.

Adjusted covariates include age (as continuous variable), gender, family history, bone metastases, and peritoneal metastases.

**Figure 3:**
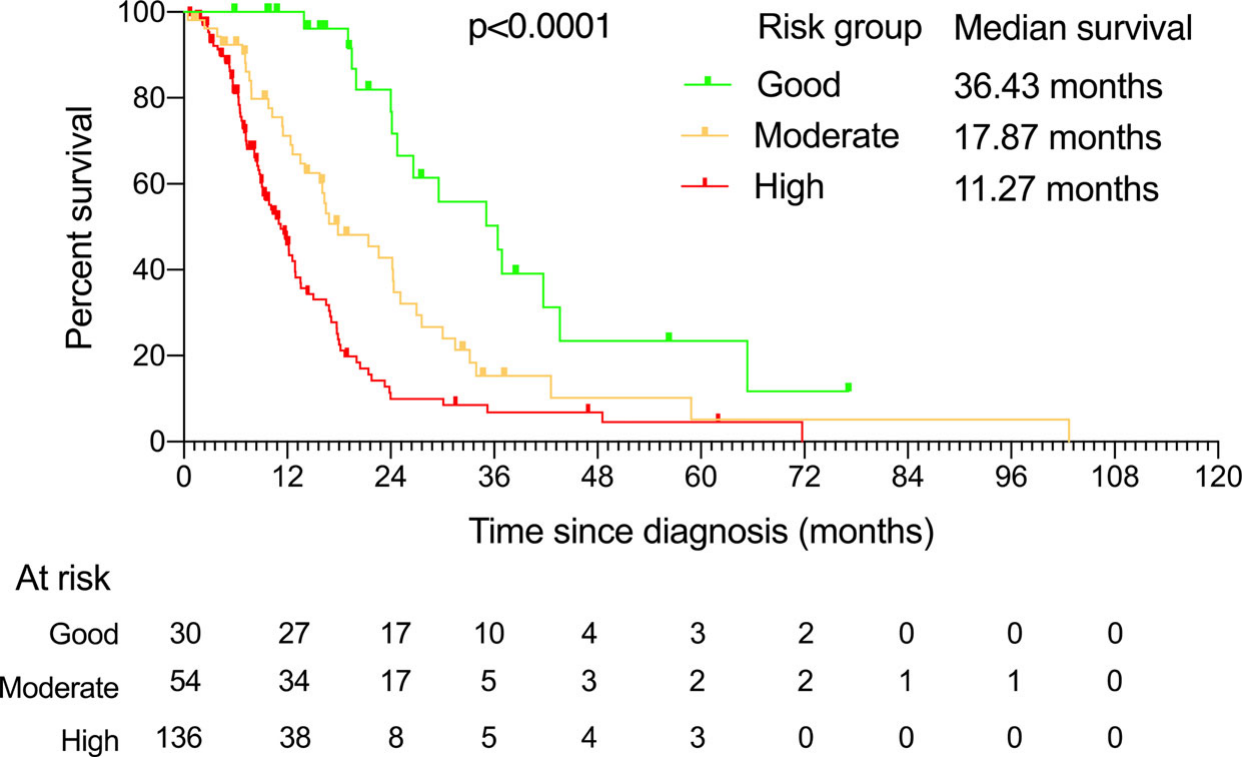
The Kaplan–Meier curves showing overall survival for each of the three risk groups determined by the prognostic factors. The median survival and the patients at risk for each of these groups are also presented.

Since the risks (as measured by HRs) of these three independent prognostic factors had a similar magnitude, except radical gastrectomy, which was counted twice due to its relative size of HR being the square of others, we then created a simple prognostic score without losing too much information for each patient by calculating the score of prognostic factors. Accordingly, the prognostic score ranged from 0 to 4 (**Table 3**). A prognostic index was devised using prognostic scores as follows: good-risk group, that is, YAAGC patients with zero to one prognostic score; intermediate-risk group, that is, YAAGC patients with two prognostic scores; and poor-risk group, that is, YAAGC patients with three to four prognostic scores. Of 220 YAAGC patients with complete data for the three variables, 30 YAAGC patients were categorized as good-risk group, 54 YAAGC patients as intermediate-risk group, and 136 YAAGC patients as poor-risk group. The Kaplan–Meier survival curves according to the prognostic model are provided in **Figure 3**. Median OS for good-, intermediate-, and poor-risk groups were 36.43 months (95% CI 22.80–49.99), 17.87 months (95% CI 10.63–25.16), and 11.27 months (95% CI 9.41–13.18), respectively. Survival differences among groups achieved statistical significance (*p* < 0.0001).

**Table 3:** Prognostic index.

Index	Score	Events	Total no. of included patients (%)
Good risk	0–1	15	30 (13.6%)
Moderate risk	2	39	54 (24.5%)
Poor risk	3–4	89	136 (61.8%)

## Discussion

In this analysis, to the best of our knowledge, we focused so far on the largest series of YAAGC. In multivariate analysis, we identified previous radical gastrectomy, serum albumin level, and NLR as significant prognostic factors. Of note, we devised a simple prognostic index for YAAGC based on easily available variables. In this model, patients in different risk groups had varying survival.

In our cohort, the positive prognostic role of previous radical gastrectomy on primary tumor is probably linked to a more favorable disease course, even though they already have had a stage IV disease. In the current knowledge, surgery is still the only chance for long-term survival for GC that can be curatively resected (7). Indeed, some reports suggested that young GC patients would benefit from curative resection or palliative debulking surgery (7, 18–20). Recently, a study by Medrano-Guzmán et al. consisting of a cohort of 588 consecutive cases supported the idea that young patients aged under 45 years who have undergone complete resection of their cancer have a better survival rate after two disease-free years, despite advanced presentation of the disease (18). Similarly, Park et al. reported a significantly higher 5-year survival rate in curatively resected young patients than older groups with GC (21). In addition, a retrospective cohort study suggested surgery as independent covariates associated with OS in young patients with non-metastatic GC (22). Furthermore, the positive status of resection margins is an unfavorable independent prognostic factor of GC in the young group (23). In fact, immediate surgery may significantly reduce the tumor burden and avoid otherwise frequent complications in YAAGC, such as obstruction, bleeding, and perforation, thus favorably affecting patient conditions and treatment tolerability (1). Given the advantage of less comorbidities, young GC patients may be better candidates to receive aggressive surgery following chemoradiation (1). Interestingly, the prognosis of young GC patients may be better than that in older patients after radical gastrectomy when matched for baseline characteristics (24). Nevertheless, whether a radical gastrectomy on primary tumor would benefit YAAGC is worth verifying in future prospective clinical research.

The NLR is a cost-effective method and a potential inflammation-based prognostic indicator for several types of cancer (16, 25, 26). In this study, NLR was an independent prognostic factor affecting the survival in YAAGC. Indeed, NLR was considered as a prognostic indicator in resectable (27), unresectable (15, 16, 28), and advanced clinical stage in GC (15, 29). NLR is also related to more aggressive tumor characteristics. In line with other study (29), NLR is associated with the occurrence of peritoneal metastases and bone metastases, as well as other markers of platelet–lymphocyte ratio (PLR) in YAAGC (**Supplementary Table 1**). This ratio thus may be used to assist in individualized follow-up and treatment (25), with a better diagnostic value than the traditional tumor markers CA19-9 and CEA (16, 29). However, we did not observe any correlation between tumor differentiation (16) and NLR for YAAGC. In contrast to previous findings (16, 29), we proposed that NLR is also a valuable predictor of prognoses in young female patients with advanced GC.

In this study, multivariate analysis indicated that hypo-albuminemia was an independent prognostic factor for YAAGC. Indeed, it is known that preoperative low serum albumin is an independent negative prognostic factor for resectable (17, 30) or advanced clinical stage in GC (31). In our cohort, a significant number of YAAGC with peritoneal metastases were included, and the accumulation of albumin in peritoneum activity may thus have a role in hypoalbuminemia. Indeed, serum albumin level was correlated to the occurrence of peritoneal metastases in YAAGC (**Supplementary Table 1**). We found that the serum albumin level was negatively correlated to both the systemic inflammatory markers NLR and PLR (**Supplementary Table 1**). Controversially, the relation between hypoalbuminemia and poor survival may be secondary to that of the systemic inflammatory response (32). Additionally, studies on the mechanism of hypoproteinemia in GC found that a massive leakage of serum albumin into the stomach occurs often in GC as well as other gastric disease (33, 34). Besides, hypoalbuminemia is reported to predict venous thromboembolism in metastatic GC patients (35) and postoperative complications after GC surgery (36). Though those are beyond the aim of this study, the relation of hypoalbuminemia and venous thromboembolisms seems a good project for a future study.

However, some limitations exist in our study. In statistical methods, dichotomization and categorization of continuous variables cause loss of information, but simplify the implementation of the analyses and interpretation of the results. In addition, the simple prognostic index based on retrospective data did require validation in an external cohort of YAAGC. Furthermore, an analysis of confounding variables may be needed to exclude possible interference in the relevant prognostic factors. Moreover, relevant histopathological parameters that affect the laboratory parameters may need to be considered for clinical application of this model.

In conclusion, three prognostic factors have been identified in young patients with advanced GC. A simple prognostic index has been developed with distinct survival rates among the different risk groups. This simple prognostic model may help in designing future trials.

## Data Availability Statement

The raw data supporting the conclusions of this article will be made available by the authors, without undue reservation.

## Ethics Statement

The studies involving human participants were reviewed and approved by Fudan University Shanghai Cancer Center (1503144-8). The patients/participants provided their written informed consent to participate in this study.

## Author Contributions

Conceptualization, data curation and collection, and manuscript review and editing: WZ and G-LC. Investigation, methodology, data analysis, and original draft preparation: G-LC and YH. Data collection and investigation: G-LC, YH, XP, W-JF, X-YZ, X-DZ, W-HL, MH, Z-YC, W-JG, and WZ. All authors contributed to the article and approved the submitted version.

## Funding

This study was supported by a grant from the National Natural Science Foundation of China (No. 81802362).

## Conflict of Interest

The authors declare that the research was conducted in the absence of any commercial or financial relationships that could be construed as a potential conflict of interest.

## Publisher’s Note

All claims expressed in this article are solely those of the authors and do not necessarily represent those of their affiliated organizations, or those of the publisher, the editors and the reviewers. Any product that may be evaluated in this article, or claim that may be made by its manufacturer, is not guaranteed or endorsed by the publisher.

## Supplementary Material

Supplementary Table 1 Correlation analysis.

## References

[1] Li J. Gastric Cancer in Young Adults: A Different Clinical Entity From Carcinogenesis to Prognosis. Gastroenterol Res Pract (2020) 2020:9512707. doi: 10.1155/2020/9512707 32190044 PMC7071806

[2] Zhou F Shi J Fang C Zou X Huang Q. Gastric Carcinomas in Young (Younger Than 40 Years) Chinese Patients: Clinicopathology, Family History, and Postresection Survival. Med (Baltimore) (2016) 95(9):e2873. doi: 10.1097/MD.0000000000002873 PMC4782856 26945372

[3] Zareba KP Zinczuk J Dawidziuk T Pryczynicz A Guzinska-Ustymowicz K Kedra B. Stomach Cancer in Young People - A Diagnostic and Therapeutic Problem. Prz Gastroenterol (2019) 14(4):283–5. doi: 10.5114/pg.2019.90254 PMC6983757 31988675

[4] Braga-Neto MB Carneiro JG de Castro Barbosa AM Silva IS Maia DC Maciel FS. Clinical Characteristics of Distal Gastric Cancer in Young Adults From Northeastern Brazil. BMC Cancer (2018) 18(1):131. doi: 10.1186/s12885-018-3995-4 29402219 PMC5800037

[5] De B Rhome R Jairam V Ozbek U Holcombe RF Buckstein M. Gastric Adenocarcinoma in Young Adult Patients: Patterns of Care and Survival in the United States. Gastric Cancer (2018) 21(6):889–99. doi: 10.1007/s10120-018-0826-x 29691758

[6] Guan WL Yuan LP Yan XL Yang DJ Qiu MZ. More Attention Should be Paid to Adult Gastric Cancer Patients Younger Than 35 Years Old: Extremely Poor Prognosis was Found. J Cancer (2019) 10(2):472–8. doi: 10.7150/jca.27517 PMC6360302 30719142

[7] Isobe T Hashimoto K Kizaki J Miyagi M Aoyagi K Koufuji K. Characteristics and Prognosis of Gastric Cancer in Young Patients. Oncol Rep (2013) 30(1):43–9. doi: 10.3892/or.2013.2467 PMC3729235 23674196

[8] Dhobi MA Wani KA Parray FQ Wani RA Wani ML Peer GQ. Gastric Cancer in Young Patients. Int J Surg Oncol (2013) 2013:981654. doi: 10.1155/2013/981654 24381753 PMC3870864

[9] Song S Li C Li S Cong X Xue Y. Clinicopathological Features and Prognoses in Younger and Older Patients With Gastric Cancer. Onco Targets Ther (2017) 10:4795–802. doi: 10.2147/OTT.S144801 PMC5628679 29033591

[10] Liu S Feng F Xu G Liu Z Tian Y Guo M. Clinicopathological Features and Prognosis of Gastric Cancer in Young Patients. BMC Cancer (2016) 16:478. doi: 10.1186/s12885-016-2489-5 27418046 PMC4946107

[11] Santoro R Carboni F Lepiane P Ettorre GM Santoro E. Clinicopathological Features and Prognosis of Gastric Cancer in Young European Adults. Br J Surg (2007) 94(6):737–42. doi: 10.1002/bjs.5600 17330827

[12] Dai F-X Jin J-J Wang W Yu S-J Long Z-W Cai H. Clinicopathological Features and Prognosis of Younger Patients With Gastric Carcinoma. Trans Cancer Res (2017) 6(2):312–21. doi: 10.21037/tcr.2017.03.19

[13] Choi YW Ahn MS Jeong GS Lee HW Jeong SH Kang SY. The Role of Surgical Resection Before Palliative Chemotherapy in Advanced Gastric Cancer. Sci Rep (2019) 9(1):4136. doi: 10.1038/s41598-019-39432-7 30858457 PMC6411914

[14] Chau I Norman AR Cunningham D Waters JS Oates J Ross PJ. Multivariate Prognostic Factor Analysis in Locally Advanced and Metastatic Esophago-Gastric Cancer–Pooled Analysis From Three Multicenter, Randomized, Controlled Trials Using Individual Patient Data. J Clin Oncol (2004) 22(12):2395–403. doi: 10.1200/JCO.2004.08.154 15197201

[15] Miyamoto R Inagawa S Sano N Tadano S Adachi S Yamamoto M. The Neutrophil-to-Lymphocyte Ratio (NLR) Predicts Short-Term and Long-Term Outcomes in Gastric Cancer Patients. Eur J Surg Oncol (2018) 44(5):607–12. doi: 10.1016/j.ejso.2018.02.003 29478743

[16] Fang T Wang Y Yin X Zhai Z Zhang Y Yang Y. Diagnostic Sensitivity of NLR and PLR in Early Diagnosis of Gastric Cancer. J Immunol Res (2020) 2020:9146042. doi: 10.1155/2020/9146042 32211444 PMC7081040

[17] Isik A Okan I Firat D Yilmaz B Akcakaya A Sahin M. A New Prognostic Strategy for Gastric Carcinoma: Albumin Level and Metastatic Lymph Node Ratio. Minerva Chir (2014) 69(3):147–53. 24970303

[18] Medrano-Guzman R Valencia-Mercado D Luna-Castillo M Garcia-Rios LE Gonzalez-Rodriguez D. Prognostic Factors for Survival in Patients With Resectable Advanced Gastric Adenocarcinoma. Cir Cir (2016) 84(6):469–76. doi: 10.1016/j.circen.2016.11.009 27039288

[19] Song P Wu L Jiang B Liu Z Cao K Guan W. Age-Specific Effects on the Prognosis After Surgery for Gastric Cancer: A SEER Population-Based Analysis. Oncotarget (2016) 7(30):48614–24. doi: 10.18632/oncotarget.9548 PMC5217043 27224925

[20] Lee J Lee MA Kim IH Roh SY. Clinical Characteristics of Young-Age Onset Gastric Cancer in Korea. BMC Gastroenterol (2016) 16:110. doi: 10.1186/s12876-016-0528-y 27600152 PMC5011834

[21] Park JC Lee YC Kim JH Kim YJ Lee SK Hyung WJ. Clinicopathological Aspects and Prognostic Value With Respect to Age: An Analysis of 3,362 Consecutive Gastric Cancer Patients. J Surg Oncol (2009) 99(7):395–401. doi: 10.1002/jso.21281 19347884

[22] Wu C Wang N Zhou H Wang T Zhao D. Development and Validation of a Nomogram to Individually Predict Survival of Young Patients With Nonmetastatic Gastric Cancer: A Retrospective Cohort Study. Saudi J Gastroenterol (2019) 25(4):236–44. doi: 10.4103/sjg.SJG_378_18 PMC6714466 30719999

[23] Hsieh FJ Wang YC Hsu JT Liu KH Yeh CN. Clinicopathological Features and Prognostic Factors of Gastric Cancer Patients Aged 40 Years or Younger. J Surg Oncol (2012) 105(3):304–9. doi: 10.1002/jso.22084 22116742

[24] Liu W Quan H Chen X Ouyang Y Xiao H. Clinicopathological Features and Prognosis of Young Gastric Cancer Patients Following Radical Gastrectomy: A Propensity Score Matching Analysis. Sci Rep (2019) 9(1):5943. doi: 10.1038/s41598-019-42406-4 30976037 PMC6459851

[25] Szor DJ Dias AR Pereira MA Ramos M Zilberstein B Cecconello I. Prognostic Role of Neutrophil/Lymphocyte Ratio in Resected Gastric Cancer: A Systematic Review and Meta-Analysis. Clinics (Sao Paulo) (2018) 73:e360. doi: 10.6061/clinics/2018/e360 29924187 PMC5996440

[26] Sun J Chen X Gao P Song Y Huang X Yang Y. Can the Neutrophil to Lymphocyte Ratio Be Used to Determine Gastric Cancer Treatment Outcomes? A Systematic Review and Meta-Analysis. Dis Markers (2016) 2016:7862469. doi: 10.1155/2016/7862469 26924872 PMC4746375

[27] Kim EY Song KY. The Preoperative and the Postoperative Neutrophil-to-Lymphocyte Ratios Both Predict Prognosis in Gastric Cancer Patients. World J Surg Oncol (2020) 18(1):293. doi: 10.1186/s12957-020-02059-4 33172490 PMC7656697

[28] Murakami Y Saito H Shimizu S Kono Y Shishido Y Miyatani K. Neutrophil-To-Lymphocyte Ratio as a Prognostic Indicator in Patients With Unresectable Gastric Cancer. Anticancer Res (2019) 39(5):2583–9. doi: 10.21873/anticanres.13381 31092456

[29] Nakamura N Kinami S Fujii Y Miura S Fujita J Kaida D. The Neutrophil/Lymphocyte Ratio as a Predictor of Peritoneal Metastasis During Staging Laparoscopy for Advanced Gastric Cancer: A Retrospective Cohort Analysis. World J Surg Oncol (2019) 17(1):108. doi: 10.1186/s12957-019-1651-3 31238937 PMC6593512

[30] Ouyang X Dang Y Zhang F Huang Q. Low Serum Albumin Correlates With Poor Survival in Gastric Cancer Patients. Clin Lab (2018) 64(3):239–45. doi: 10.7754/Clin.Lab.2017.170804 29739107

[31] Huamán MO Cerna-Barco J Correa-López LE Beltran-Garate B de la Cruz -Vargas JA. Albumina E Índice Neutrófilo-Linfocito Como Predictores De Estadío Tumoral En Pacientes Con Cáncer Gástrico. Rev la Facultad Med Hum (2020) 20(2):96–113. doi: 10.25176/RFMH.v20i2.2936

[32] Crumley AB Stuart RC McKernan M McMillan DC. Is Hypoalbuminemia an Independent Prognostic Factor in Patients With Gastric Cancer? World J Surg (2010) 34(10):2393–8. doi: 10.1007/s00268-010-0641-y 20602101

[33] Glass GBJ Ishimori A. Passage of Serum Albumin Into the Stomach. Am J Digest Dis (1961) 6(2):103–33. doi: 10.1007/BF02231798 13705938

[34] Jarnum S Schwartz M. Hypoalbuminemia in Gastric Carcinoma. Gastroenterology (1960) 38:769–76. doi: 10.1016/S0016-5085(60)80091-1 14406821

[35] Takayoshi K Kusaba H Aikawa T Koreishi S Sagara K Nakano M. Hypoalbuminemia for the Prediction of Venous Thromboembolism and Treatment of Direct Oral Anticoagulants in Metastatic Gastric Cancer Patients. Gastric Cancer (2019) 22(5):988–98. doi: 10.1007/s10120-019-00930-2 30788749

[36] Zhou J Hiki N Mine S Kumagai K Ida S Jiang X. Role of Prealbumin as a Powerful and Simple Index for Predicting Postoperative Complications After Gastric Cancer Surgery. Ann Surg Oncol (2017) 24(2):510–7. doi: 10.1245/s10434-016-5548-x 27638673

