# Supplementary material for: Three-Tier Prognostic Index in Young Adults With Advanced Gastric Cancer
Source: Front Oncol. 2021 Sep 10;11:667655. doi: 10.3389/fonc.2021.667655 (PMC8462089; doi:10.3389/fonc.2021.667655)
Supplement: Supplementary Table 1 — Correlation analysis. [file Table_1.docx]

Supplementary Material

Supplementary Table 1, correlation analysis

| Variables | | Pearson Correlation | P value |
| --- | --- | --- | --- |
| Gender | Liver metastases | 0.275 | 0.000 |
| Neutrophil-lymphocyte ratio | Platelets-lymphocyte ratio | 0.409 | 0.000 |
|  | Peritoneal metastases | 0.177 | 0.009 |
|  | Bone metastases | 0.191 | 0.004 |
| Albumin | Neutrophil-lymphocyte ratio | -0.352 | 0.000 |
|  | Platelets-lymphocyte ratio | -0.349 | 0.000 |
| Albumin < 40 g/L | Peritoneal metastases | 0.196 | 0.004 |
